# Epidemiologic Features and Influencing Factors of Norovirus Outbreaks in the City of Wuxi, China from 2014 to 2018

**DOI:** 10.4269/ajtmh.20-1371

**Published:** 2021-10-18

**Authors:** Qian Geng, Yuan Shen, Ping Shi, Yu-Meng Gao, Wei-Hong Feng, Yong Xiao, Xiaoying Ma, Shanshan Xie, KeWei Wang, Jie Gao, Chao Shi

**Affiliations:** ^1^ Department of Disease Control, Wuxi Center for Disease Control and Prevention, Wuxi, Jiangsu, China;; ^2^ Department of Public Health, Health Supervision Institute of Xinwu District, Wuxi, Jiangsu, China;; ^3^ Department of Microorganism Labs, Wuxi Center for Disease Control and Prevention, Wuxi, Jiangsu, China;; ^4^ Affiliated Hospital of Jiangnan University, Wuxi, Jiangsu, China;; ^5^ Department of Infection Control, Shanghai Children’s Hospital, Shanghai Jiaotong University, Shanghai, China

## Abstract

The study investigated the genotypic changes and epidemiologic features of norovirus outbreaks and factors influencing the attack rate and outbreak duration in Wuxi from 2014 to 2018. Norovirus outbreaks, monitored through surveillance system, were investigated. The norovirus-positive specimens from outbreaks were collected and genotyped using a dual polymerase-capsid genotyping protocol based on a one-step polymerase chain reaction (PCR) amplicon. The genotypes were analyzed by Norovirus Typing Tool Version 2.0. A total of 74 norovirus outbreaks were reported in Wuxi from 2014 to 2018. Most (93.2%) norovirus outbreaks were caused by GII genotypes. The predominant norovirus genotypes in outbreaks have changed from GII.17 (20.3%) in 2014–2015 to GII.P16/GII.2 (40.5%) in 2017–2018. GII.P16/GII.2 in 2017–2018 season were more prevalent than GII.17 in 2014–2015 season (χ^2^ = 4.741, *P* = 0.029). 56.7% of the outbreaks occurred in primary schools. The re-outbreak rate was 16.2%. 66.7% of re-outbreaks were caused by norovirus variants different from previous genotypes. Outbreaks in nonprimary school settings (odds ratio [OR]: 4.007; 95% CI: 1.247–12.876) and those leading to temporary school or institution closure (OR: 20.510; 95% CI: 1.806–232.937) were reported with a higher attack rate. The outbreaks in primary schools (OR: 4.248; 95% CI: 1.211–14.903), re-outbreaks (OR: 6.433; 95% CI: 1.103–37.534) and longer report timing (OR: 8.380; 95% CI: 2.259–31.089) declared a significantly longer duration. It is of great importance that the monitoring of norovirus outbreaks for the emergence of novel strains, along with responsive prevention and control intervention should be strengthened in adults and school-age population, especially in primary students and preschool children.

## INTRODUCTION

Norovirus is a genetically diverse virus that belongs to the family Caliciviridae.^1^ The viral genome consists of a single positive-strand RNA of 7.7 kb encompassing three open reading frames (ORFs). The ORF1 of norovirus genome encodes six nonstructural proteins including the RNA-dependent RNA polymerase (RdRp). ORF2 and ORF3 encode the major capsid protein VP1 and minor capsid protein VP2, respectively. On the basis of amino acid identified in VP1, norovirus is classified into seven genogroups (GI-GVII) of which GI, GII, and GIV are responsible for human infections. To date, norovirus has been genetically categorized into 39 genotypes, with at least nine genotypes for GI and 22 genotypes for GII.^2^ In the past few years, the circulation of norovirus GII genogroups has increased significantly both in developed countries^3^^–^^7^ and developing countries.^8^^–^^11^

Norovirus infection may induce symptoms such as vomiting, diarrhea, abdominal pain, mild fever and nausea in infected cases.^12^ As a leading global cause of diarrheal diseases across all ages, norovirus infection was estimated to account for 18% of acute gastroenteritis (AGE) cases and at least 50% of gastroenteritis outbreaks worldwide.^13^^,^^14^ Due to the low infectious dose and stable survival in the environment, noroviruses are highly contagious, and thus, it easily results in large outbreaks with high attack rate.^15^ In addition, norovirus re-outbreaks may occur as a result of host’s short-lasting immunity and long duration of viral shedding.^16^

Wuxi is a well-developed city in the southeast of Jiangsu Province, China. It has five districts and two counties with a total population of 6.5 million people. In 2009, Wuxi launched a laboratory-based gastroenteritis surveillance program. Similar to other studies in China,^17^ norovirus was reported as the most common pathogen of AGE illnesses in Wuxi, with a proportion of 58.9% (data not shown) in AGE outbreaks. Previous studies in other cities have described that the circulating genotypes of norovirus infection changed from GII.4 to new variants GII.P16/GII.2 in China,^9^^,^^18^^–^^22^ however, little information is available on the genotypic trends and epidemiologic features of norovirus outbreaks in Wuxi. In this study, we analyzed the genotypes shift and explored the influencing factors associated with attack rate and duration of norovirus outbreaks in Wuxi.

## MATERIALS AND METHODS

### Surveillance of gastroenteritis outbreaks.

Outbreaks of norovirus gastroenteritis in Wuxi were monitored through two surveillance systems. The first system is the Emergent Public Health Event Information Management System, in which an acute gastroenteritis outbreak is defined as ≥ 20 cases with symptoms including vomiting and/or diarrhea within 1 week. The other system is the norovirus outbreak surveillance system in Jiangsu province, in which an outbreak is defined as 5–19 cases with symptoms of vomiting and/or diarrhea within 3 days. Each outbreak must be reported to Wuxi Municipal CDC (Wuxi CDC). Outbreak reports included data and information such as year, month, type of exposure settings, number of cases, number of exposed persons, sanitation status, control measures, onset of first and last case, as well as reporting date of outbreak. On the basis of the outbreaks scale, the control measures were categorized into case isolation, unit closure, and temporary school or institution closure. Case isolation meant that cases could not go to school or work till their recovery. Case isolation was conducted in work institutions or school facilities with ≤ 25% infections in the same class or work department. Unit closure meant that the classroom or work department was temporarily closed. Unit closure was conducted in work institutions or school facilities with > 25% infections in the same classroom or work department. Temporary school or institution closure meant that the school or work institution was temporarily closed for the outbreak. Temporary school or institution closure was conducted in the situation with ≥ 25% infections in the whole school or work institution.

### Sample collection, norovirus detection, and genotyping.

Feces or vomitus specimens from outbreaks were tested for noroviruses by Wuxi CDC. The RNA extraction and norovirus detection were conducted as previously described.^23^ For each norovirus-positive outbreak, one norovirus-positive sample was selected for genotyping using a dual polymerase-capsid genotyping protocol based on a one-step polymerase chain reaction (PCR) amplicon obtained with primer pair Mon431/G2SKR for GII strains and Mon432/G1SKR for GI strains (Table 1). The genotypes were determined by Norovirus Typing Tool Version 2.0 (https://www.rivm.nl/mpf/typ-ingtool/norovirus).

**Table 1:** Primers used in one-step RT-PCR

Premiers	Sequence	Polarity
Mon431	TGG ACI AGR GGI CCY AAY CA	+
G2SKR	CCR CCN GCA TRHCCR TTR TAC AT	−
Mon432	TGG ACI CGY GGI CCY AAY CA	+
G1SKR	CCA ACC CAR CCATTR TAC A	−

RT-PCR = reverse transcription polymerase chain reaction.

### Re-outbreaks screening criteria.

The screening criteria for re-outbreaks included 1) the name of exposure setting was the same one; and 2) > 14 days between the two reporting dates of outbreaks.

### Statistical analysis.

Descriptive statistics were used to analyze the epidemiological characteristics of outbreaks. Categorical variables were presented as numbers and proportions, and continuous variables as median and interquartile range (IQR). The χ^2^ test was applied to compare the proportion of GII.P16/GII.2 and GII.17 in their epidemic seasons. The Kruskal–Wallis H test and Mann–Whitney *U* test were conducted for stratified comparisons of attack rate and outbreak duration. Influencing factors for norovirus attack rate and outbreak duration were assessed using logistic regression model. Variables significant in univariate logistic regression were included into multivariate regression model. All analysis was performed with SPSS version 16.0 (SPSS, Chicago, IL). *P* values were derived from two-tailed tests and significant was assumed for *P* < 0.05.

### Ethical considerations.

This investigation was performed in response to a public health emergency, and based on the Regulation on the Urgent Handling of Public Health Emergencies (http://www.gov.cn/zwgk/2005-05/20/content_145.htm), formal ethical approval was not required. But verbal consent was obtained from all participants before the interview and sampling. Parents or guardians of participants under 15 years granted consent on their behalf and accompanied them during the interview. Consent was recorded on the questionnaire using the participant’s and/or guardian’s signature. All participants were informed of their rights according to the law outlined above. We can confirm that all data, including all questionnaires and samples, were gathered in accordance with the “Guideline for norovirus outbreak reports and investigation,” issued by the Health Department of Jiangsu Province, China. No additional data were acquired by the authors, and no participant identifying information was associated with the reported data.

## RESULTS

### The changes in circulating genotypes.

**Figure 1.**
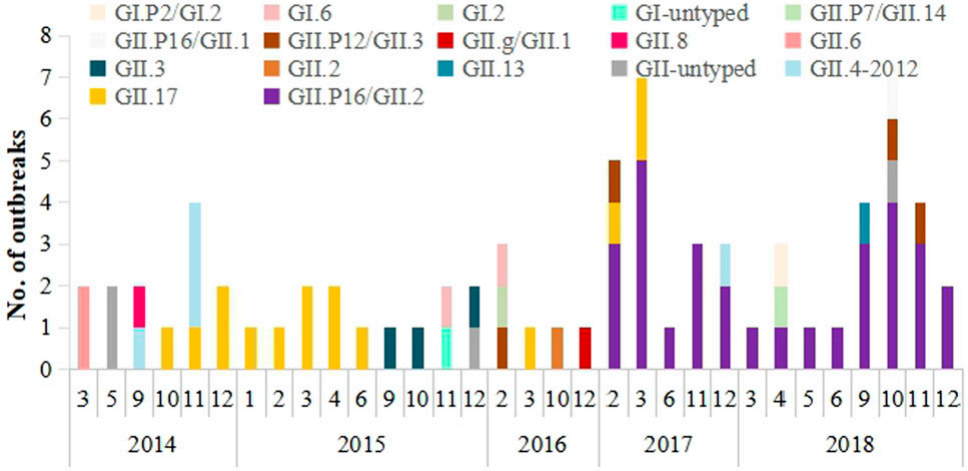
The changes of circulating genotypes detected in norovirus outbreaks in Wuxi, 2014–2018. This figure appears in color at www.ajtmh.org.

From January 2014 to December 2018, a total of 74 norovirus outbreaks were reported in Wuxi. All outbreaks were genotyped into 17 variants, including four GI variants and 13 GII variants. The GII variants were responsible for 93.2% (*N* = 69) of norovirus outbreaks. Variants GII.P16/GII.2 and GII.17 were the two major circulating genotypes during the study period, accounting for 40.5% and 20.3% of the outbreaks, respectively. Other genotypes detected in the study can be seen in Figure 1.

The variant GII.17 was first detected in October 2014 and it circulated in 2014–2017. The proportion of GII.17 outbreaks was 42.3% (11/26) in 2014–2015 season, and then significantly decreased to 16.0% (4/25) in 2016–2017 season (χ^2^ = 4.249, *P* = 0.039). The variant GII.P16/GII.2 emerged in February 2017 and resulted in a sharp increase of outbreaks in early spring 2017 and autumn in 2017–2018. This variant caused 71.4% (30/42) of outbreaks in 2017–2018 season, which presented higher epidemic than the predominance of GII.17 in 2014–2015 season (χ^2^ = 4.741, *P* = 0.029).

### The epidemiologic features and characteristics on outbreak-associated indicators.

All the 74 outbreaks were associated with 2,564 illness and 114,099 exposed people. The overall median attack rate was 2.1% (IQR: 1.1–4.1%) and overall median outbreak duration was 5.0 days (IQR: 3.0–7.0). The reported outbreaks and outbreak-associated indicators in different groups were listed in Table 2. The majority (70.3%, *N* = 52) of outbreaks were reported in spring and autumn. About 85.1% (*N* = 63) of outbreaks occurred in urban area. Most (73.0%, *N* = 54) of outbreaks occurred in settings with good sanitation status. However, there was no significant difference in both attack rate and outbreak duration among seasons, locations, genotypes, and sanitation status, respectively (*P* > 0.05). The outbreaks most frequently reported in primary schools (56.7%, *N* = 42), followed by preschool facilities (20.3%, *N* = 15), secondary schools (17.6%, *N* = 13) and settings involving adults and elderly people (5.4%, *N* = 4). The attack rate and outbreak duration varied by exposure settings (*P* < 0.05). By contrast to outbreak frequency order, the attack rate was highest in settings involving adults and elderly people (11.3%, IQR: 4.5–18.2%), and lowest in primary school (1.7%, IQR: 0.9–2.8%). The outbreak duration was longest both in settings involving adults and elderly people (6.5 days, IQR: 1.0–15.0 days) and primary school (6.5 days, IQR: 3.0–9.3 days), and shortest in preschool facilities (3.0 days, IQR: 2.0–4.0 days). 67.6% (*N* = 50) of outbreaks were reported within 3 days since the onset of index illness. The duration of outbreaks reported within 3 days was statistically shorter than that reported at least 4 days (4.0 days, IQR: 2.0–7.0 days versus 7.0 days, IQR: 5.0–13.5 days, *P* < 0.001). Control measures were intervened into all outbreaks, with unit closure accounting for 62.2% (*N* = 46). The attack rate of outbreaks with unit closure was lowest among the various control measures (*P* = 0.006). Temporary school or institution closure was conducted when the attack rate of at that time reached 2.9% (IQR: 1.4–4.1%), and unit closure was initiated when attack rate reached 1.0% (IQR: 0.7–2.0%).

**Table 2:** The reported outbreaks and outbreak-associated indicators in different groups in Wuxi, 2014–2018

Outbreaks characteristics	No. of outbreaks (%)	Attack rate		Outbreak duration	
		Median (IQR)	*P*	Median (IQR)	*P*
Season			0.637		0.783
Spring	22 (29.7%)	2.0 (1.1–5.9)		5.0 (2.0–9.3)	
Summer	3 (4.1%)	1.5 (0.4–3.0)		5.0 (2.0–6.0)	
Autumn	30 (40.5%)	2.0 (0.8–4.2)		5.0 (3.0–7.0)	
Winter	19 (25.7%)	2.2 (1.3–3.8)		5.0 (3.0–13.0)	
Location			0.755		0.339
Urban	63 (85.1%)	2.1 (1.0–4.1)		5.0 (3.0–10.0)	
Rural	11 (14.9%)	2.0 (1.3–3.0)		5.0 (2.0–6.0)	
Type of exposure settings			0.004†		0.001†
Preschool facilities	15 (20.3%)	2.9 (1.4–8.6)		3.0 (2.0–4.0)	
Primary schools	42 (56.7%)	1.7 (0.9–2.8)		6.5 (3.0–9.3)	
Secondary schools	13 (17.6%)	3.0 (1.2–4.3)		6.0 (4.5–16.0)	
Institution involving adults and elderly people*	4 (5.4%)	11.3 (4.5–18.2)		6.5 (1.0–15.0)	
Genotypes			0.575		0.203
GII.P16/GII.2	30 (40.5%)	2.1 (1.1–4.1)		6.5 (4.0–12.0)	
GII.17	15 (20.3%)	2.2 (1.3–6.6)		5.0 (1.0–12.0)	
Other genotypes	29 (39.2%)	2.0 (0.9–3.2)		5.0 (3.0–7.0)	
Report timing§			0.982		< 0.001‡
1–3 days	50 (67.6%)	2.0 (1.1–4.0)		4.0 (2.0–7.0)	
4–12 days	24 (32.4%)	2.2 (1.1–4.1)		7.0 (5.0–13.5)	
Sanitation status			0.068		0.093
Good	54 (73.0%)	2.0 (1.0–3.4)		5.0 (3.0–9.3)	
Poor	20 (27.0%)	3.0 (1.1–8.6)		3.5 (2.5–6.8)	
Control measures			0.006†		0.167
Case isolation	15 (20.3%)	2.2 (1.2–3.8)		3.0 (2.0–10.0)	
Unit closure	46 (62.2%)	1.7 (1.0–3.2)		5.0 (3.0–7.0)	
Temporary school or institution closure	13 (17.6%)	4.1 (2.7–5.9)		5.0 (3.5–13.5)	
Total	74 (100%)	2.1 (1.1–4.1)		5.0 (3.0–7.0)	

IQR = interquartile range.

* Institution involving adults and elderly people included three companies and one nursing home.

† The comparison of attack rate and outbreak duration were conducted by the Kruskal–Wallis H test.

‡ The comparison of outbreak duration was conducted by Mann–Whitney *U* test.

§ Report timing indicated the time interval between the onset of first case and outbreak online report.

### The norovirus re-outbreaks.

Of 62 exposure settings reporting 74 outbreaks in all, eight settings, namely preschool facilities and schools, experienced 12 re-outbreaks in total. The re-outbreak rate was 16.2% (12/74). Six settings reported norovirus outbreaks twice (8.1%), one setting three times (2.7%), and one setting five times (5.4%). In settings that reported outbreaks twice, the time interval between two outbreaks was 8.5 (IQR: 3.3–29.3) months. In settings that reported outbreaks three times, the time intervals of outbreaks were 9 months and 32 months. In the setting that reported outbreaks five times, the time intervals of outbreaks were 12 months, 6 months, 12 months, and 23 months, respectively. About 75% of re-outbreaks occurred within 12 months.

Of 12 re-outbreaks, 33.3% (*N* = 4) were caused by a common GII.P16/GII.2 in two outbreaks, 8.3% (*N* = 1) were caused by GII.17 in primary outbreak and GII.P16/GII.2 in re-outbreaks. About 25.1% (*N* = 3) were caused by other genotypes in primary outbreak and GII.P16/GII.2 in re-outbreaks. The proportion of GII.P16/GII.2 detected in re-outbreaks was as high as 75.0% (Table 3).

**Table 3:** The genotypes detected in primary infection and secondary infection

Primary outbreak	Re-outbreaks	No. of outbreaks (%)
GII.P16/GII.2	GII.P16/GII.2	4 (33.3%)
GII.17	GII.P16/GII.2	1 (8.3%)
	Other genotypes	1 (8.3%)
Other genotypes	GII.P16/GII.2	3 (25.1%)
	GII.17	1 (8.3%)
	Other genotypes	2 (16.7%)

### The influencing factors for norovirus attack rate and outbreak duration.

The univariate (Table 4) and multivariate logistic regression model indicated that the type of exposure settings and control measures were found to have a significant influence for norovirus attack rate, with nonprimary school settings and temporary school or institution closure being risk factors for a relatively higher attack rate (Table 5). In addition, the logistic regression model demonstrated that the type of exposure settings, re-outbreaks and report timing were the influencing factors for norovirus outbreak duration, with the outbreaks in primary schools, re-outbreaks and longer report timing (4–12 days) declaring a relatively longer outbreak duration (Table 6).

**Table 4:** Univariate logistic regression analysis on influencing factors for norovirus attack rate and outbreak duration

Factors	Attack rate				Duration outbreak			
	β	wald	OR (95% CI)	*P*	β	wald	OR (95% CI)	*P*
Season								
Spring	–	–	1	–	–	–	–	–
Summer	−0.511	0.155	0.600 (0.047–7.630)	0.694	−0.454	0.506	0.635 (0.182–2.219)	0.477
Autumn	−0.086	0.565	0.918 (0.303–2.776)	0.879	−0.588	0.202	0.556 (0.043–7.214)	0.653
Winter	0.721	1.27	2.057 (0.587–7.211)	0.26	−0.028	0.002	0.972 (0.307–3.074)	0.962
Location								
Urban	–	–	1	–	–	–	1	–
Rural	−0.151	0.053	0.860 (0.238–3.111)	0.818	−0.336	0.245	0.2714 (0.290–2.688)	0.619
Type of exposure settings								
Primary schools	–	–	1	–	–	–	1	–
Nonprimary school settings	1.234	6.291	3.436 (1.310–9.016)	0.012	−1.129	5.087	0.323 (0.121–0.862)	0.24
Genotypes								
GII.P16/GII.2	–	–	1	–	–	–	1	–
GII.17	0.203	0.101	1.224 (0.351–4.269)	0.751	−0.775	2.098	0.461 (0.161–1.315)	0.147
Other Genotypes	−0.065	0.015	0.938 (0.337–2.606)	0.902	−0.539	0.706	0.583 (0.166–2.052)	0.401
Re-outbreaks								
Yes	−1.228	2.96	0.293 (0.072–1.186)	0.085	2.207	7.267	9.091 (1.827–45.246)	0.007
No	–	–	1	–	–	1	–	–
Report timing								
1–3 days	–	–	1	–	–	–	1	–
4–12 days	0.327	0.432	1.387 (0.522–3.684)	0.511	1.735	10.134	5.667 (1.948–16.487)	0.001
Sanitation status								
Good	–	–	1	–	–	–	1	–
Poor	0.918	2.849	2.503 (0.862–7.264)	0.091	−0.773	1.914	0.462 (0.154–1.380)	0.166
Control measures								
Case isolation	−3.114	8.221	0.44 (0.005–0.373)	0.004	0.067	0.011	1.069 (0.317–0.675)	0.915
Unit closure	−2.351	4.092	0.095 (0.010–0.930)	0.043	−0.857	1.13	0.424 (0.087–2.061)	0.288
Temporary school or institution closure	–	–	1	–	–	–	1	–

**Table 5:** Multivariate logistic regression analysis on influencing factors on attack rate of norovirus outbreaks

Factors	Β	wald	OR (95% CI)	*P*
Type of exposure settings				
Primary schools	–	–	1	–
Nonprimary school settings	1.388	5.433	4.007 (1.247–12.876)	0.02
Control measures				
Case isolation	–	–	1	–
Unit closure	−0.112	0.686	0.894 (0.233–3.431)	0.871
Temporary school or institution closure	3.021	5.937	20.510 (1.806–232.937)	0.015

**Table 6:** Multivariate logistic regression analysis on influencing factors on norovirus outbreak duration

Factors	Β	wald	OR (95% CI)	*P*
Type of exposure settings				
Primary schools	1.447	5.103	4.248 (1.211–14.903)	0.024
Nonprimary school settings	–	–	1	–
Re-outbreaks				
Yes	1.861	4.279	6.433 (1.103–37.534)	0.039
No	–	–	1	–
Report timing				
1–3 days	–	–	1	–
4–12 days	2.126	10.1	8.380 (2.259–31.089)	0.001

## DISCUSSION

Norovirus is highly genetically diverse and constantly evolving, resulting in the emergence of new genotypes every 2–4 years.^24^^,^^25^ From January 2014 to December 2018, a total of 74 outbreaks were reported in Wuxi, and noroviruses were presented in 17 genotypes. Consistent with other findings in China,^9^ norovirus GII was the most commonly detected strains in Wuxi. And the prevalence of various genotypes in this study was in accordance with the general evolvement of norovirus GII in the rest of the world.^26^^–^^28^ In our study, the genotypic changes of norovirus outbreaks were characterized into three distinct phases. The first phase showed a prevalence of GII.17 from October 2014 to June 2015. The GII.17 variant was firstly reported in September 2014 in Asia.^29^ This variant quickly became the predominant in other continents, raising a global concern on its pandemics.^26^^,^^30^^,^^31^ Before the emergence of GII.17, GII.4 was the most major circulating genotype worldwide in norovirus outbreaks.^4^^,^^5^^,^^7^ However, GII.4 was presented as a minor strain in our study and mainly detected in 2014 autumn season, indicating a possible shift from GII.4 to GII.17 in late 2014. In the second phase, no dominant genotypes were identified and a diversity of GI and GII variants cocirculated during September 2015 to December 2016. The coexistence of multiple genotypes may facilitate the potential variant evolvement, which would increase the number of acute norovirus outbreaks. During 2017–2018 season, namely the last phase, novel recombinant GII.P16/GII.2 prevailed with an overwhelming predominance. In agreement with other published studies,^18^^,^^32^ GII.P16/GII.2 displayed a higher epidemic than GII.17, resulting in a steep rise of norovirus outbreaks in 2017. This new recombinant emerged in February 2017 in Wuxi, which was later than the time reported in other studies. Previously, GII.P16/GII.2 was reported to firstly appear in August 2016 in China and rapidly resulted in dozens of outbreaks in different areas.^33^^,^^34^ Likewise, in Jiangsu Province, GII.P16/GII.2 was firstly detected in December 2016 and spread extensively across the province.^32^ Based on the earlier findings, we could speculate that the circulation of GII.P16/GII.2 in Wuxi was introduced from other neighboring cities.

Norovirus outbreaks in China generally peaked in the winter and early spring.^17^ Similarly, the remarkable increase of norovirus outbreaks could be observed from autumn to early spring season in our study, which was possibly related to the low temperature and high humidity. Schools, including primary and secondary schools, and preschool facilities accounted for 94.6% of all the outbreaks in Wuxi. A similar percentage of norovirus outbreaks among schools and childcare facilities was also reported on a nation-level research in China.^17^ Schools, as well as preschool facilities, seemed to be more densely populated than other countries because of a large capacity of nearly 50 students in each classroom. The high contact rate in students and their insufficient immunity to the virus could increase the possibility of morbidity, especially among children with poor personal hygiene.^35^ Our descriptive analysis indicated that the outbreaks in school settings and preschool facilities were associated with relatively lower attack rate than those in institutions involving adults and elderly people. This result may be attributed to the high reporting frequency of norovirus illnesses in schools and preschool facilities, which strengthened the rapid emergency response and disease control. Additionally, the routine check and health screening in children attending kindergarten and schools was conducted each morning to exclude cases with fever, vomiting, or diarrhea. The program has been helpful to discover the suspected norovirus illnesses and reduce the potential infection and transmission. For measures on disease control, the intervention, including case isolation, unit closure, and temporary school or institution closure, would be introduced in terms of outbreaks severity. The outbreaks intervened with temporary school or institution closure were scientifically reported with large number of norovirus-associated illnesses, and thus, they were reported with a significantly greater attack rate. Furthermore, our results showed a shorter duration among outbreaks reported within 3 days, compared with outbreaks reported 4 days later, underlining a beneficial effect on the responsive outbreak report to local CDC.

Prolonged viral shedding from both symptomatic and asymptomatic infections, combined with limited long-term immunity, greatly contributed to secondary virus spread and norovirus re-outbreak.^36^ It was observed that norovirus re-outbreaks with various genotypes occurred frequently in children^37^ and that re-outbreaks could occur within a year in both children and adults.^38^^,^^39^ In our analysis, the majority of re-outbreaks were also reported in primary schools and preschool facilities, namely young children. Also, a high percentage (75%) of re-outbreaks occurred within 12 months. Another significant finding was that re-outbreaks with different norovirus genotypes were common. This could be because of a limited direct immune protection for the same genotypes.

The influencing factors on norovirus outbreaks attack rate and duration were not well researched yet, because published studies were mostly analyzed on a single reported outbreak rather than a cluster of outbreaks in one area. In this study, multivariate analysis indicated that outbreaks occurred in nonprimary school settings and that with temporary school or institution closure experienced a statistically higher attack rate. Moreover, our results demonstrated that norovirus outbreak duration were significantly associated with the type of exposure settings, re-outbreaks and report timing, with the outbreaks in primary schools, re-outbreaks and longer report timing (4–12 days) declaring a relatively longer duration. Therefore, the monitoring of norovirus outbreaks, along with responsive prevention and control interventions, should be strengthened in adults and school-age population, particularly among primary students and preschool children. Targeted health education promotion should be conducted in primary schools for developing a strong preventive consciousness of norovirus infection.

There were several limitations in this study. Firstly, epidemiological information collected by local CDCs was limited, to some extent. Route of transmission, as well as gender and age information are not required for outbreak reports, which may be useful for identifying the risk factors and appropriate control measures. In addition, the results were inevitably subjected to inaccuracy of outbreak data. The attack rates and outbreak duration were probably underestimated because of underreporting. Secondly, asymptomatically infected individuals, as well as close-contacts such as family/sibling relationship, were excluded from our study, which could have impacted the outcomes of norovirus epidemiologic features. Thirdly, the multilevel modeling/variability analysis for both individuals and groups was not implemented in our study.

## CONCLUSION

Although a diversity of norovirus genotypes was observed in Wuxi, the reported norovirus outbreaks were presented with an alternating predominance of GII.17 in 2014–2015 and GII.P16/GII.2 in 2017–2018. The majority of re-outbreaks were reported in primary schools and preschool facilities. In comparison to primary outbreak, re-outbreaks were frequently caused by different norovirus genotypes. The outbreaks occurred in nonprimary school settings and those with temporary school or institution closure experienced a higher attack rate. And the outbreaks duration in primary schools, re-outbreaks and longer report timing (4–12 days) were relatively longer. It is critical that the monitoring of norovirus outbreaks, as well as responsive prevention and control interventions, should be strengthened in adults and school-age population, especially in primary students and preschool children.
